# Sociodemographic inequities in unscheduled asthma care visits among public assistance recipients in Japan: additional risk by household composition among workers

**DOI:** 10.1186/s12913-023-10110-9

**Published:** 2023-10-11

**Authors:** Daisuke Nishioka, Junko Saito, Keiko Ueno, Naoki Kondo

**Affiliations:** 1 Department of Medical Statistics, Osaka Medical and Pharmaceutical University, Research & Development Center, 2-7 Daigaku-Machi, Takatsuki-Shi, Osaka, 569-8686 Japan; 2 Department of Social Epidemiology, Graduate School of Medicine and School of Public Health, Kyoto University, Yoshida-Konoe-Cho, Sakyo-Ku, Kyoto, Kyoto Japan; 3 Division of Behavioral Sciences, National Cancer Center Institute for Cancer Control, 5-1-1 Tsukiji, Chuo-Ku, Tokyo, Japan; 4 Institute for Future Initiatives, the University of Tokyo, 7-3-1, Hongo, Bunkyo-Ku, Tokyo, Japan; 5 Japan Agency for Gerontological Evaluation Study (JAGES Agency), 6-3-5 Yanaka, Taito-Ku, Tokyo, Japan

**Keywords:** Asthma, Poverty, Unscheduled visit, Public assistance, Japan

## Abstract

**Background:**

Public assistance programs aim to prevent financial poverty by guaranteeing a minimum income for basic needs, including medical care. However, time poverty also matters, especially in the medical care adherence of people with chronic diseases. This study aimed to examine the association between the dual burden of working and household responsibilities, with unscheduled asthma care visits among public assistance recipients in Japan.

**Methods:**

This retrospective cohort study included public assistance recipients from two municipalities. We obtained participants’ sociodemographic data in January 2016 from the public assistance database and identified the incidence of asthma care visits. Participants’ unscheduled asthma visits and the frequency of asthma visits were used as the outcome variables. Unscheduled visits were defined as visits by recipients who did not receive asthma care during the first three months of the observation period. Participants’ age, sex, household composition, and work status were used as explanatory variables. Multiple Poisson regression analyses were performed to calculate the cumulative incidence ratio (IR) with a 95% confidence interval (CI) of unscheduled visits across the explanatory variables. The effect of modification on the work status by household composition was also examined.

**Results:**

We identified 2,386 recipients at risk of having unscheduled visits, among which 121 patients (5.1%) had unscheduled visits. The multivariable Poisson regression revealed that the working recipients had a higher incidence of unscheduled visits than the non-working recipients (IR 1.44, 95% CI 1.00–2.07). Among working recipients, the IRs of unscheduled visits were higher among recipients cohabiting with adults (IR 1.90 95% CI 1.00–3.59) and with children (IR 2.35, 95% CI 1.11–4.95) than for recipients living alone. Among non-working recipients, the IRs of unscheduled visits were lower for recipients living with family (IR 0.74, 95% CI 0.41–1.35) and those living with children (IR 0.50, 95% CI 0.20–1.23). A higher frequency in asthma visits was observed among working recipients living with family.

**Conclusions:**

Working adults cohabiting with children are at the greatest risk of unscheduled visits among adults receiving public assistance. To support healthy lifestyles of public assistance recipients, medical care providers and policymakers should pay special attention to the potentially underserved populations.

**Supplementary Information:**

The online version contains supplementary material available at 10.1186/s12913-023-10110-9.

## Background

Interest in preventing the exacerbation of asthma symptoms is increasing [1]. Otherwise, these symptoms may lower patients’ quality of life, decrease productivity and opportunities, and increase social costs [2]. Asthma is an ambulatory care sensitive condition, [3, 4] a chronic health condition for which appropriate interventions in primary care can prevent exacerbations and hospital admissions. Therefore, encouraging patients’ continuity of asthma care that prevents its aggravation can be crucial from the primary care and public health perspectives.

Exacerbation of asthma symptoms can be prevented by appropriate continuous care; [4, 5] however, such care is often interrupted for several reasons [6–9]. For example, some patients find it difficult to recognize the need to receive care during asymptomatic periods. Patients need to pay for direct or indirect medical care and opportunity costs for care consultations. Patients’ low health literacy can lead to misunderstandings regarding care services, and the loss of general care further leads to unscheduled asthma care visits, [10] causing exacerbation of asthma symptoms [11].

Asthma is more prevalent among the impoverished population, [12] or those who may be at a greater risk for unscheduled visits owing to the cost burden of asthma care. Therefore, ensuring financial access to asthma care among the impoverished population is crucial. To support the healthy lives of the impoverished population, many countries have introduced governmental welfare programs such as public assistance, [13] called *“seikatsu-hogo”* in Japan [14].

Public assistance can provide financial and medical support to those below the poverty line. The eligibility for public assistance is based on family members’ demographic characteristics and the socioeconomic conditions of their residential areas. For example, to be eligible for public assistance, a person living alone in Tokyo must have an income of approximately less than 120,000 JPY and have no other assets [15, 16]. Approximately 1.6% of the Japanese population receives public assistance and monthly minimum income protection [14]. All public assistance recipients can avail of the medical assistance program, which exempts recipients from out-of-pocket payments for medical care. After receiving public assistance, according to the Public Assistance Act, “A public assistance recipient shall constantly work diligently, make efforts to reduce his/her expenditure, and make other efforts to maintain and improve his/her standard of living” [16]. Municipal welfare offices encourage recipients to work to become self-sufficient and not rely on public assistance.

Although recipients benefit from minimum income protection and assured financial health care access, their frequent visits to healthcare facilities have been discussed as a policy issue for quite some time. Living alone or not working have been reported as significant predictors of frequent visits to health care facilities [17, 18]. Certainly, the behavioral characteristics of poor people and multidimensional poverty (e.g., social isolation, psychosocial stress) can affect recipients’ health care utilization. Impoverished populations also face time poverty, which is characterized by insufficient discretionary time—the time available after engaging in necessary physiological and committed activities of paid and unpaid work, such as employment, household duties, or parenting [19]. All these factors affect the adherence to asthma care by public assistance recipients, leading to unscheduled visits and the exacerbation of asthma symptoms [9, 11, 12, 20].

However, the potential risk factors of having unscheduled visits among public assistance recipients are not well investigated. To fill this gap, we examined the association between individual non-financial sociodemographic factors leading to time constraints and unscheduled asthma visits among adult public assistance recipients using public assistance recipient databases administered by two municipalities in Japan.

## Materials and methods

### Study design

This is a retrospective cohort study.

### Study participants

This study included adults aged 20–64 years who received public assistance in two suburban municipalities in Osaka and Tokyo in January 2016. We excluded individuals who became self-sufficient and no longer relied on public assistance during the observation period and those who received long-term care.

### Data sources

We used the public assistance recipient database of municipal welfare offices. This database included information on age, sex, number of family members, household composition, nationality, and work status. As the data collected by the municipality welfare offices determined the public assistance eligibility and the amount of monthly income protection, there were no missing data. To obtain the outcome data, we used administrative medical assistance claims data from January to December 2016, including the month of the recipients’ medical consultation, the total cost of medical receipts, the total number of visits each month, and their diagnosis. This database did not provide information on whether the recipient’s asthma consultation was for preventive or treatment purposes (e.g., prescriptions or medical treatment).

Each municipality individually linked the two databases using individual identification codes. The welfare offices of both municipalities agreed to provide anonymized data to the authors via a company system, which provided municipalities with the management software of the public assistance database. The study protocol was approved by the Ethics Committee of the Kyoto University Graduate School and Faculty of Medicine (approval no: R3356). Further, all methods were carried out in accordance with relevant guidelines and regulations of the Japanese governments [21, 22] and Declaration of Helsinki.

### Measurement and variables

#### Outcome variable

We used recipients’ unscheduled asthma visits as the outcome variable. Unscheduled visits refer to asthma care visits by recipients who did not receive asthma care during the first three months of the observation period. To identify unscheduled visits, we first determined from the medical assistance claims data the cumulative incidence of asthma consultation among all recipients, defined as those who received asthma care at least once during the observation period. Cumulative incidence is the total number of cases of disease among all individuals in the population at risk during the observation period [23]. The definition of asthma was determined according to the International Classification of Diseases, Tenth Edition (ICD-10) code J45-46. To identify the recipients with a potential risk of unscheduled visits (i.e., the population at risk of unscheduled visits), we recognized recipients who had asthma consultations during the first three months of the observation period (i.e., between January and March 2016). We assumed that these recipients received scheduled asthma care because, in Japan, scheduled asthma consultation for preventive purposes usually occurs within three months [24]. Although these populations might include recipients engaged in unscheduled visits, excluding them can also eliminate the potential reverse causation between the explanatory variables and unscheduled asthma visits (e.g., those living alone or those not working because of severe asthma, which led to receiving public assistance).

#### Explanatory variables

Household composition (i.e., living alone, living with children under 15 years old [living with children], not living alone, and not living with children [living with adults]), work status (working or not), and nationality (Japanese or others) as of January 2016, were used as sociodemographic variables.

#### Covariates

Age (continuous) and sex (women/men) were included as biological covariates. Furthermore, we used certificates for mental, intellectual, and physical disabilities, noting the information on the qualifications for welfare benefits for disabled persons because the recipients of disability assistance can benefit from additional income and social care. Municipality officials certified them for the diagnoses by designated physicians and agencies. We coded the municipality as a dummy variable to adjust for unmeasured cultural and environmental characteristics of the two municipalities (A/B).

### Statistical analysis

First, we described the participants’ characteristics that led to a greater risk of having unscheduled visits. Next, we performed univariable and multivariable Poisson regression analyses and calculated crude and adjusted cumulative incidence ratios (IR) of asthma consultation and the 95% confidence interval (CI) of each explanatory variable among populations at risk of having unscheduled visits. We additionally performed analyses stratified by work status to consider the impact of time constraints on healthcare access among recipients who engaged in work and household duties. Further, to verify the adherence and behavioral patterns of recipients’ medical visits, we reconsidered the outcome as count-data on the frequency of visits during the observation period using a Poisson regression analysis.

We performed three sensitivity analyses. First, we described the characteristics of recipients with scheduled visits and those with unscheduled visits, and conducted a stratified analysis by work status. Second, we included an interaction term of work status and household composition into the multiple regression model to examine the robustness of our findings. Third, we performed the original analyses by redefining unscheduled visits as those of recipients who did not receive asthma care during the first four months of the observation period. This allows examining the robustness and validity of the definition of scheduled/unscheduled visits and variance in patients’ consultation behavior. A robust standard error estimator was adopted for all statistical analyses to calculate the 95% CI. Statistical analyses were performed using STATA MP Ver.18 (Stata Corp., College Station, TX, USA).

## Results

**Fig. 1:**
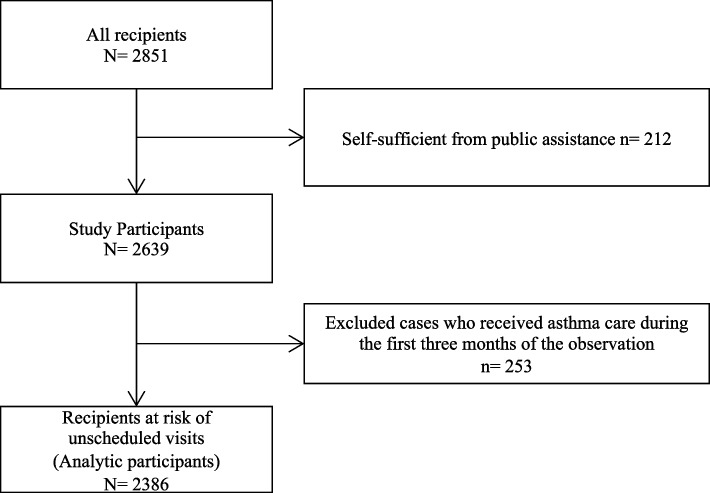
Flowchart of study participants

We obtained data from 2,639 public assistance recipients. During the first three months of the study period, 253 recipients had asthma consultations. After excluding these recipients, we identified 2,386 recipients at risk of unscheduled visits (Fig. 1). The population comprised 1,261 (52.8%) men, 1,452 (60.9%) recipients living alone, 279 recipients living with children (11.7%), 757 (31.7%) working recipients, and 121 recipients (5.2%) engaged in unscheduled visits (Table 1). Recipients who had unscheduled visits were more likely to be working than those who had scheduled visits (41.3% vs 30.4%, *p* = 0.04; Supplementary Table S1). Among working recipients, those living with children were more likely to have unscheduled visits, and among non-working recipients, those living alone were more likely to have unscheduled visits (Supplementary Table S2).

**Table 1:** Unscheduled asthma care visits: public assistance recipient characteristics and visit incidence across variables

		Population at risk of unscheduled visits (*N* = 2,386)	Unscheduled visits (*n* = 121, 5.1% of N)
Characteristic	Category	N (%)	n, % for N
Age	Mean (SD)	48.5 (11.1)	45.0 (10.9)
Sex	Male	1,261 (52.8)	47, 3.7%
	Female	1,125 (47.2)	74, 6.6%
Household composition	Living alone	1,452 (60.9)	65, 4.5%
	Living with children	279 (11.7)	19, 7.0%
	Living with adults	655 (27.4)	37, 5.6%
Work status	Working	757 (31.7)	50, 6.6%
	Not working	1,629 (68.3)	71, 4.4%
Nationality	Japanese	2,285 (95.8)	116, 5.1%
	Other	101 (4.2)	5, 5.0%
Disabilities certificate	None	1,790 (75.0)	85, 4.7%
	Mental disability	373 (15.6)	24, 6.4%
	Intellectual disability	71 (3.0)	3, 4.2%
	Physical disability	152 (6.4)	9, 5.9%
Municipality	A	1,797 (75.3)	92, 5.1%
	B	589 (24.7)	29, 4.9%

The results of the multivariable Poisson regression analyses (Model 1) showed that the adjusted IR by age (by 10-year increments) was 0.79 (95% CI, 0.68–0.93). Female recipients showed a higher IR of unscheduled visits than male recipients (IR 1.64; 95% CI 1.13–2.38). Working recipients showed a higher IR of unscheduled visits than non-working recipients (IR 1.44; 95% CI, 1.00–2.07). No significant differences were observed across household compositions (Table 2).

**Table 2:** Public assistance recipients’ unscheduled asthma care visits: crude and adjusted incidence ratios and 95% confidence intervals

		Crude	Adjusted
Characteristic	Category	IR, (95% CI)	IR, (95% CI)
*Explanatory variables*			
Work status	Not working	Ref	Ref
	Working	1.52 (1.07–2.15)	1.44 (1.00–2.07)
Household composition	Living alone	Ref	Ref
	Living with children	1.52 (0.93–2.5)	0.99 (0.58–1.70)
	Living with adults	1.26 (0.85–1.87)	1.03 (0.67–1.57)
Nationality	Japanese	Ref	Ref
	Other	0.98 (0.41–2.33)	0.97 (0.41–2.28)
*Covariates*			
Age	by 10 years	0.77 (0.67–0.88)	0.79 (0.68–0.93)
Sex	Male	Ref	Ref
	Female	1.76 (1.24–2.52)	1.64 (1.13–2.38)
Disabilities certificate	None	Ref	Ref
	Mental disability	1.35 (0.87–2.1)	1.47 (0.92–2.33)
	Intellectual disability	0.89 (0.29–2.75)	0.72 (0.22–2.33)
	Physical disability	1.25 (0.64–2.43)	1.67 (0.85–3.26)
Municipality	A	Ref	Ref
	B	0.96 (0.64–1.44)	0.97 (0.65–1.45)

IR means incidence ratios and CI means confidence interval

Multivariable Poisson regression analyses stratified by work status showed an effect modification of the association of work status with unscheduled visits by household compositions. Stronger effect modification was found among recipients living with children (Table 3). Among working recipients, the IRs of unscheduled visits were higher among recipients living with adults (IR 1.90, 95% CI 1.00–3.59) and those living with children (IR 2.35, 95% CI 1.11–4.95) than among recipients living alone. Conversely, among non-working recipients, the IRs of unscheduled visits were lower for recipients living with adults (IR 0.74, 95% CI 0.41–1.35) and those living with children (IR 0.50 95% CI 0.20–1.23). The sensitivity analysis showed consistent results (see Supplementary Table S3 and S4).

**Table 3:** Unscheduled asthma care visits: adjusted incidence ratios and 95% confidence intervals, stratified by work status

		Working Recipients (*n* = 757)	Non-working Recipients (*n* = 1,629)
		Adjusted	Adjusted
Characteristic	Category	IR, (95% CI)	IR, (95% CI)
*Explanatory variables*			
Household composition	Living alone	Ref	Ref
	Living with children	2.35 (1.11–4.95)	0.50 (0.20–1.23)
	Living with adults	1.90 (1.00–3.59)	0.74 (0.41–1.35)
Nationality	Japanese	Ref	Ref
	Other	0.90 (0.30–2.70)	0.87 (0.22–3.51)
*Covariates*			
Age	by 10 years	0.94 (0.71–1.24)	0.71 (0.59–0.86)
Sex	Male	Ref	Ref
	Female	1.34 (0.76–2.36)	1.90 (1.18–3.06)
Disabilities certificate	None	Ref	Ref
	Mental disability	2.12 (0.99–4.54)	1.21 (0.68–2.15)
	Intellectual disability	0.60 (0.08–4.32)	1.15 (0.28–4.73)
	Physical disability	2.92 (0.98–8.67)	1.39 (0.60–3.21)
Municipality	A	Ref	Ref
	B	0.49 (0.20–1.19)	1.28 (0.79–2.10)

IR means incidence ratios and CI means confidence interval

When reconsidering the outcome as count-data on the frequency of visits during the observation period, the multivariable Poisson regression analyses showed that the frequency tended to be higher among recipients living with adults (IR 1.39, 95% CI 0.92–2.10) and those living with children (IR 1.46, 95% CI 0.85–2.51) than among recipients living alone (Table 4). Meanwhile among non-working recipients, the frequency was not strongly associated with the recipients’ household composition (living with adults 1.19, 95% CI 0.89–1.58; living with children 1.01 95% CI 0.65–1.56; Table 4).

**Table 4:** Frequency of asthma visits: adjusted incidence ratios and 95% confidence intervals, stratified by work status

		Working Recipients (*n* = 757)	Non-working Recipients (*n* = 1,629)
		Adjusted	Adjusted
Characteristic	Category	IR, (95% CI)	IR, (95% CI)
*Explanatory variables*			
Household composition	Living alone	Ref	Ref
	Living with children	1.46 (0.85–2.51)	1.01 (0.65–1.56)
	Living with adults	1.39 (0.92–2.1)	1.19 (0.89–1.58)
Nationality	Japanese	Ref	Ref
	Other	0.67 (0.27–1.65)	0.67 (0.3–1.5)
*Covariates*			
Age	by 10 years	1.05 (0.88–1.25)	0.94 (0.84–1.07)
Sex	Male	Ref	Ref
	Female	1.38 (0.94–2.02)	2.16 (1.64–2.83)
Disabilities certificate	None	Ref	Ref
	Mental disability	1.75 (1.05–2.89)	1.11 (0.8–1.53)
	Intellectual disability	0.89 (0.32–2.49)	0.89 (0.36–2.17)
	Physical disability	1.34 (0.49–3.69)	1.26 (0.79–1.99)
Municipality	A	Ref	Ref
	B	0.69 (0.41–1.16)	1.13 (0.85–1.49)

IR means incidence ratios and CI means confidence interval

## Discussion

Among public assistance recipients in Japan, the risk of unscheduled asthma visits was higher among young people, women, and working recipients. An effect modification of work status on unscheduled visits by household composition was investigated, especially among recipients living with children. The frequency of asthma visits was slightly higher among working recipients living with family members. This is the first study to depict the prevalence of asthma among public assistance recipients and demonstrate an association between the incidence of unscheduled asthma visits and individual sociodemographic factors among public assistance recipients in Japan. We identified the population likely to be underserved from usual asthma care and to engage in unscheduled asthma visits despite having financial security for minimum living income and medical costs.

### Interpretation

Working individuals in Japan are susceptible to missing out on their scheduled asthma care [6] Our study added to this evidence by demonstrating that the risk of having unscheduled visits by working individuals was amplified by cohabitation with family members in impoverished populations despite financial aid. The impoverished population receiving public assistance is expected to become self-sufficient (i.e., work and earn money for daily living). The results show that working recipients living with their families were more likely to have unscheduled visits and frequent medical visits for asthma care. This phenomenon can be explained by the adherence practices of such recipients. Previous studies have shown that being employed and having responsibilities at home (caring for children) are socioeconomic determinants of medical care adherence [25–27]. Further, most of the working recipients were non-regular workers [18, 19]. Non-regular workers in Japan are more likely to work in environments where benefits such as medical checkups are not established [28]. Non-regular workers experience difficulty in health care visits because absence from work leads to a loss of income, which they want to avoid. Recipients who rear their children report that they lack sufficient time to receive medical checkups and go for health care visits; [29] this qualitative evidence further supports the findings of this study.

### Implications

A community-based primary care approach from medical care providers may effectively address the risk of having unscheduled visits [30, 31]. Medical care providers caring for patients with asthma should ameliorate public assistance recipients’ social risk patterns, such as working/living with children, to prevent unscheduled asthma care visits. After identifying a patient’s social risks in primary care, providing collaborative care with social care sectors, such as welfare offices and other voluntary organizations, can reduce the risk of unscheduled visits (e.g., social prescribing and patient navigation) [32, 33]. In addition, primary care providers can collaborate with professionals working on occupational health and health management in the workplace to prevent recipients from missing scheduled consultations.

### Strengths and limitations

An important strength of this study is that by using existing standardized databases without missing data, we were able to examine the association between individuals’ sociodemographic backgrounds and unscheduled asthma visits among socially vulnerable populations who are usually difficult to access in standard social surveys. Furthermore, our study suggested an effect modification of work status on unscheduled visits by household composition, especially for those living with children, despite financial aid for daily living and medical care among adult public assistance recipients.

However, this study has several limitations. First, as this study used medical assistance claims data, we may have overestimated or underestimated the incidence of asthma consultations. Such consultations only occur when recipients visit medical facilities. Owing to the characteristic of the public assistance system in Japan, some recipients do not avail of the medical assistance programs. For example, if recipients are receiving other types of financial support for health care (e.g., welfare support for severe mental disabilities or diseases designated by the government as intractable), their consultations are not reflected in the medical assistance claims data. Physicians assign specific diagnoses for insurance claims data. However, the diagnosis of asthma can be influenced by several factors such as the physician’s clinical sub-specialty, medical examinations, and the prescribed medications. Because the data we used in this study did not include information regarding prescriptions or medical treatments, and no validation study has been conducted on such data for asthma care, potential misclassifications may occur. Hence, further work is needed. Second, it is possible that we misclassified patients engaged in unscheduled visits during the first three months of the observation period as having scheduled visits. If so, this would have created a crucial bias to the study findings if there were more older people, men, non-working recipients, and recipients living alone who engaged in unscheduled visits. However, our findings suggest that those likely to engage in unscheduled visits were young people, women, working recipients, and recipients living with family members, and our results are consistent with those of previous studies that focused on the care adherence of recipients with chronic health conditions [25, 26, 34, 35]. The results of the sensitivity analyses using the modified period also support the study findings. Therefore, we can assume that such a misclassification, if any, did not create a bias sufficient to overturn our conclusions. Third, this study did not evaluate other risk factors for asthma adherence, such as smoking behavior, types of paid and unpaid work, educational attainment, or social relationships, which may also affect adherence to asthma care. In addition, other unmeasured factors, such as the severity of the disease and degree of medical treatment, may lead to potential bias in our findings. Fourth, generalizability is limited as this study used data on public assistance recipients from two municipalities in Japan.

## Conclusions

Although minimum income and financial access to medical care are ensured, working recipients living with family members, especially children, may be at greater risk of having unscheduled asthma visits because of non-adherence to asthma care. Further studies with more socioeconomic and behavioral information are necessary. Nevertheless, our study suggests that medical care providers and policymakers should pay special attention to these potentially underserved populations to support their healthy lives.

### Supplementary Information

**Additional file 1: Table S1.** Comparison of recipient characteristics: those with scheduled visits versus those with unscheduled visits. **Table S2.** Comparison of recipient characteristics: those with scheduled visits versus those with unscheduled visits, stratified by work status. **Table S3.** Public assistance recipients’ unscheduled asthma care visits: adjusted incidence ratios and 95% confidence intervals: an interaction model. **Table S4.** Modified unscheduled visits: adjusted incidence ratios and 95% confidence intervals, stratified by work status. 

## Data Availability

The data used in this study was obtained from the participating municipalities in Japan; however, there are restrictions regarding the availability of this data as it was used under license for the current study and is not publicly available. The author’s data is available upon reasonable request with permission from the municipalities. Please contact the corresponding author for any requests.

## Abbreviations

CI: Confidence interval
IR: Incidence ratio
JAGES: Japan Agency for Gerontological Evaluation Study
