# Supplementary material for: Sociodemographic inequities in unscheduled asthma care visits among public assistance recipients in Japan: additional risk by household composition among workers
Source: BMC Health Serv Res. 2023 Oct 11;23:1084. doi: 10.1186/s12913-023-10110-9 (PMC10568886; doi:10.1186/s12913-023-10110-9)
Supplement: Supplementary file 1 — Additional file 1: Table S1. Comparison of recipient characteristics: those with scheduled visits versus those with unscheduled visits. Table S2. Comparison of recipient characteristics: those with scheduled visits versus those with unscheduled visits, stratified by work status. Table S3. Public assistance recipients’ unscheduled asthma care visits: adjusted incidence ratios and 95% confidence intervals: an interaction model. Table S4. Modified unscheduled visits: adjusted incidence ratios and 95% confidence intervals, stratified by work status. [file 12913_2023_10110_MOESM1_ESM.docx]

Table S1. Comparison of recipient characteristics: those with scheduled visits versus those with unscheduled visits.

|  |  |  | Scheduled visits (n=253) | Unscheduled visits (n=121) |  |
| --- | --- | --- | --- | --- | --- |
| Characteristic | Category |  | N (%) | n, % for N | p-value |
| Age | Mean (SD) |  | 48.7 (10.7) | 45.0 (10.9) | 0.002 |
| Sex |  |  |  |  | 0.32 |
|  | Male |  | 85 (33.6%) | 47 (38.8%) |  |
|  | Female |  | 168 (66.4%) | 74 (61.2%) |  |
| Household composition | |  |  |  | 0.70 |
|  | Living alone |  | 130 (51.4%) | 65 (53.7%) |  |
|  | Living with children |  | 35 (13.8%) | 19 (15.7%) |  |
|  | Living with adults |  | 88 (34.8%) | 37 (30.6%) |  |
| Work status |  |  |  |  | 0.038 |
|  | Working |  | 77 (30.4%) | 50 (41.3%) |  |
|  | Not working |  | 176 (69.6%) | 71 (58.7%) |  |
| Nationality |  |  |  |  | 0.35 |
|  | Japanese |  | 247 (97.6%) | 116 (95.9%) |  |
|  | Other |  | 6 (2.4%) | 5 (4.1%) |  |
| Disabilities certificate | |  |  |  | 0.93 |
|  | None |  | 186 (73.5%) | 85 (70.2%) |  |
|  | Mental disability |  | 45 (17.8%) | 24 (19.8%) |  |
|  | Intellectual disability |  | 6 (2.4%) | 3 (2.5%) |  |
|  | Physical disability |  | 16 (6.3%) | 9 (7.4%) |  |
| Municipality |  |  |  |  | 0.84 |
|  | A |  | 190 (75.1%) | 92 (76.0%) |  |
|  | B |  | 63 (24.9%) | 29 (24.0%) |  |

Table S2. Comparison of recipient characteristics: those with scheduled visits versus those with unscheduled visits, stratified by work status.

|  |  | Working recipients | |  | Non-working recipients | |  |
| --- | --- | --- | --- | --- | --- | --- | --- |
| Characteristic | Category | Scheduled visits (n=77) | Unscheduled visits (n=50) | p-value | Scheduled visits (n=176) | Unscheduled visits (n=71) | p-value |
| Age | Mean (SD) | 47.8 (10.6) | 44.3(12.7) | 0.094 | 49.0(10.7) | 45.5(9.47) | 0.017 |
| Sex |  |  |  | 0.85 |  |  | 0.18 |
|  | Male | 29 (38%) | 18 (36%) |  | 56 (32%) | 29 (41%) |  |
|  | Female | 48 (62%) | 32 (64%) |  | 120 (68%) | 42 (59%) |  |
| Household composition | |  |  | 0.09 |  |  | 0.11 |
|  | Living alone | 39 (51%) | 18 (36%) |  | 91 (52%) | 47 (66%) |  |
|  | Living with adults | 26 (34%) | 19 (38%) |  | 62 (35%) | 18 (25%) |  |
|  | Living with children | 12 (16%) | 13 (26%) |  | 23 (13%) | 6 (9%) |  |
| Nationality |  |  |  | 0.34 |  |  | 0.8 |
|  | Japanese | 75 (97%) | 47 (94%) |  | 172 (98%) | 69 (97%) |  |
|  | Other | 2 (3%) | 3 (6%) |  | 4 (2%) | 2 (3%) |  |
| Disabilities certificate | |  |  |  | 0.47 |  |  |
|  | None | 61 (79%) | 38 (76%) |  | 125 (71%) | 47 (66%) |  |
|  | Mental disability | 12 (16%) | 8 (16%) |  | 33 (19%) | 16 (23%) |  |
|  | Intellectual disability | 3 (4%) | 1 (2%) |  | 3 (2%) | 2 (3%) |  |
|  | Physical disability | 1 (1%) | 3 (6%) |  | 15 (8%) | 6 (9%) |  |
| Municipality |  |  |  | 0.37 |  |  | 0.46 |
|  | A | 65 (84%) | 45 (90%) |  | 125 (71%) | 47 (66%) |  |
|  | B | 12 (16%) | 5 (10%) |  | 51 (29%) | 24 (34%) |  |

| Table S3. Public assistance recipients’ unscheduled asthma care visits: adjusted incidence ratios and 95% confidence intervals: an interaction model. | | | |
| --- | --- | --- | --- |
|  |  |  | Adjusted |
| Characteristic | Category |  | IR, (95% CI) |
| *Explanatory variables* | |  |  |
| Work status | |  |  |
|  | Not working |  | Ref |
|  | Working |  | 1.01 (0.58-1.75) |
| Household composition | |  |  |
|  | Living alone |  | Ref |
|  | Living with children |  | 0.62 (0.26-1.49) |
|  | Living with adults |  | 0.81 (0.46-1.44) |
| Work status x Household composition | |  |  |
|  | Working x Living alone |  | Ref |
|  | Working x Living with children |  | 2.60 (0.88-7.68) |
|  | Working x Other |  | 1.83 (0.80-4.17) |
| Nationality |  |  |  |
|  | Japanese |  | Ref |
|  | Other |  | 0.92 (0.39-2.16) |
| *Covariates* |  |  |  |
| Age | by 10 years |  | 0.79 (0.67-0.93) |
| Sex |  |  |  |
|  | Male |  | Ref |
|  | Female |  | 1.65 (1.13-2.39) |
| Disabilities certificate | |  |  |
|  | None |  | Ref |
|  | Psychological disability |  | 1.49 (0.93-2.37) |
|  | Intellectual disability |  | 0.75 (0.23-2.41) |
|  | Physical disability |  | 1.65 (0.84-3.22) |
| Municipality |  |  |  |
|  | A |  | Ref |
|  | B |  | 0.94 (0.63-1.42) |

Note: IR means incidence ratios and CI means confidence interval.

| Table S4. Modified unscheduled visits: adjusted incidence ratios and 95% confidence intervals, stratified by work status. | | | | | |
| --- | --- | --- | --- | --- | --- |
|  |  |  | Working Recipients | | Non-working Recipients |
|  |  |  | Adjusted |  | Adjusted |
| Characteristic | Category |  | IR, (95% CI) |  | IR, (95% CI) |
| Explanatory variables | |  |  |  |  |
| Age | by 10 year |  | 0.90 (0.67-1.2) | | 0.67 (0.54-0.82) |
| Sex |  |  |  |  |  |
|  | Male |  | Ref |  | Ref |
|  | Female |  | 1.28 (0.71-2.32) | | 2.02 (1.2-3.38) |
| Household composition | |  |  |  |  |
|  | Living alone |  | Ref |  | Ref |
|  | Living with children | | 2.21 (1.05-4.66) | | 0.57 (0.29-1.12) |
|  | Other |  | 1.76 (0.92-3.36) | | 0.51 (0.21-1.27) |
| Nationality |  |  |  |  |  |
|  | Japanese |  | Ref |  | Ref |
|  | Other |  | 1.01 (0.34-3.02) | | 1.05 (0.26-4.22) |
| Covariates |  |  |  |  |  |
| Disabilities certificate | |  |  |  |  |
|  | None |  | Ref |  | Ref |
|  | Psychological disability | | 2.00 (0.89-4.5) |  | 1.33 (0.73-2.45) |
|  | Intellectual disability | | 0.61 (0.08-4.38) | | 1.40 (0.34-5.86) |
|  | Physical disability | | 3.08 (1.04-9.18) | | 1.14 (0.41-3.16) |
| Municipality |  |  |  |  |  |
|  | A |  | Ref |  | Ref |
|  | B |  | 0.54 (0.22-1.31) |  | 1.18 (0.7-2.01) |

Note: Modified unscheduled visits mean unscheduled visits excluding the cases with consultation in the first four months. IR means incidence ratios and CI means confidence interval.
